# A Review of NDT/Structural Health Monitoring Techniques for Hot Gas Components in Gas Turbines

**DOI:** 10.3390/s19030711

**Published:** 2019-02-09

**Authors:** Frank Mevissen, Michele Meo

**Affiliations:** Materials Research Centre, Department of Mechanical Engineering, University of Bath, Bath BA2 7AY, UK; fm548@bath.ac.uk

**Keywords:** gas turbines, SHM, non-destructive testing (NDT), sensing

## Abstract

The need for non-destructive testing/structural health monitoring (SHM) is becoming increasingly important for gas turbine manufacturers. Incipient cracks have to be detected before catastrophic events occur. With respect to condition-based maintenance, the complex and expensive parts should be used as long as their performance or integrity is not compromised. In this study, the main failure modes of turbines are reported. In particular, we focus on the turbine blades, turbine vanes and the transition ducts of the combustion chambers. The existing monitoring techniques for these components, with their own particular advantages and disadvantages, are summarised in this review. In addition to the vibrational approach, tip timing technology is the most used technique for blade monitoring. Several sensor types are appropriate for the extreme conditions in a gas turbine, but besides tip timing, other technologies are also very promising for future NDT/SHM applications. For static parts, like turbine vanes and the transition ducts of the combustion chambers, different monitoring possibilities are identified and discussed.

## 1. Introduction

The components of a gas turbine, in direct contact with hot gas, are subject to high loads. These include turbine blades that have to withstand extreme conditions. Monitoring the health of these components is important for two reasons. First, it is important to detect incipient cracks before catastrophic events occur, and second, it is the basis for condition-based maintenance. 

Structural health monitoring (SHM) is a further development of non-destructive testing and evaluation (NDT&E). Years ago, it seemed to be sufficient to test components before use or again in a disassembled machine. The vision is moving in the direction of realizing component damage detection in real time. Once the damage occurs, a warning is issued and information about the remaining life of the affected component is displayed. This leads to increased operating safety of the machine and at the same time reduces maintenance costs and time. Therefore, an optimal utilisation of these parts from the customer point of view is highly desirable.

An exemplary SHM system consists of many components. Transducers are needed for actuation and sensing. A signal generator supplies the actuator with diagnostic input while the sensor carries the measured data to the data acquisition system. A NDT/SHM system requires appropriate transducers, its signal processing system to reduce noise, consider environmental conditions and write down the damage signature. In order to implement this practically, knowledge of typical damage experienced by the structures are required. This can be a physical model of the structure, a statistical model or machine learning schemes [1].

Proulx published a book on SHM of rotating machines focused on vibration methods [2]. In [3,4,5] new sensor technologies and trends in SHM are reported.

This review summarises the different failure modes, which are relevant for NDT/SHM in industrial gas turbines and currently used monitoring technologies. In particular, critical defects are discussed and how to determine the remaining useful life of the turbine components. For the component load it makes a big difference whether the machine is started up once and stays in this state for a long time or whether permanent load changes take place. Depending on these operating modes, the life limiting factors for the highly loaded turbine components are shown.

Furthermore it is indicated, which consequences small cracks on turbine blades can have. The subsequent turbine stages up to the entire machine can be severely damaged. A challenge is certainly the massive, forged turbine discs where the blades are mounted. For example, at overspeed, it must be ensured that the centrifugal force always bursts the blades first before the discs do. Only in this way can it be ensured that no rotor components break through the outer casing of the gas turbine.

**Figure 1:**
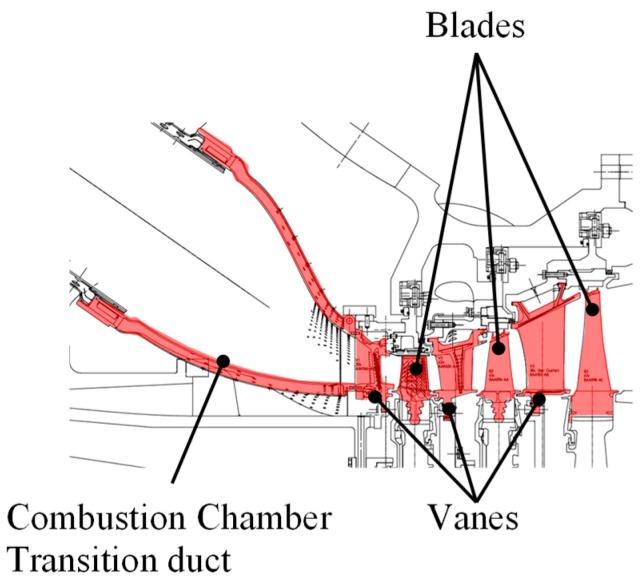
Gas turbine–Combustion chamber transition duct, turbine vanes and blades.

We also review the different monitoring techniques for gas turbine blades, vanes and combustion chamber transition ducts (Figure 1). For the static parts (vanes and transition ducts), the same monitoring techniques could be used. 

Strain gauges were used to obtain information about the components during machine operation. However, these have some significant disadvantages. With the tip timing method, sensors are placed opposite the rotating blades in the casing. These can usually determine both the important gap between blade and casing and the dynamic behaviour of the blades in-situ. To implement this technically, there are a number of sensor technologies, which are presented in more detail. This principle is already well established in turbomachinery, but there are some promising alternatives that are based on very different concepts. These monitoring techniques can set the course for a shift away from scheduled-based maintenance.

The physics of the different techniques are described in this review. Also the accuracy, the sensitivity and the bandwidth of the sensors are explained. Due to the limited installation space in the turbine area, the sizes of the sensors are also important and are evaluated. A particular challenge is the high ambient temperature, in which the sensors must work. The limits are shown below. Qualitatively the sensor costs are also evaluated. The hot gas components, in particular, have complex designs and are expensive. The great competition and price pressure in this business segment makes it necessary to offer cost-optimized series machines and signal processing units. Relevant research examples with the general areas of application are shown.

## 2. Failure Modes 

Continuous duty machines are typically applied in power generation. Therefore, an electric generator will be driven at a relatively constant speed.

Cyclic duty means mechanical drive applications where a gas turbine drives another turbomachine (e.g., a compressor). The speed of the gas turbine is adjusted to requirements of the driven unit. Due to this different operating behaviour, different failure modes can occur. The typical failure modes for hot gas parts in a gas turbine in continuous duty and cyclic duty machines are listed in Table 1 [6,7,8]. 

For peaking machines, thermal mechanical fatigue (TMF) is the main life limiting failure mode. For continuous duty machines, creep, oxidation and corrosion are the main life limiters [7].

Low-cycle and high-cycle fatigue are primary influenced by design issues, while foreign object damage and corrosion are load and environment dependent [9].

**Figure 2:**
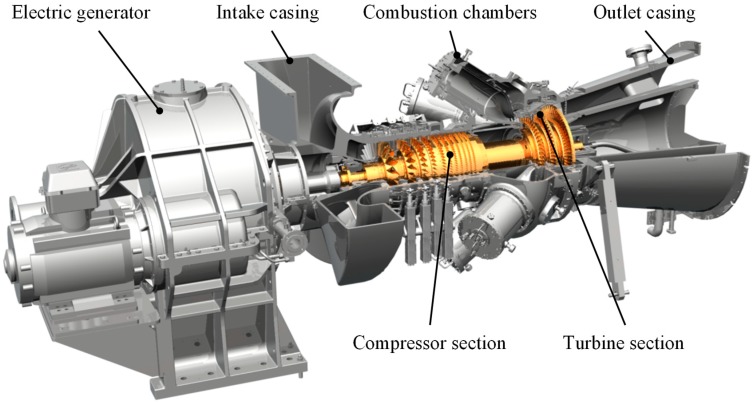
Typical gas turbine construction.

One typical failure mode for the transition ducts of combustion chambers is wear at the mating surfaces. High temperature gradients, especially at transient operating conditions, allow the parts to rub against each other. Another failure mode is the degradation of the thermal barrier coating (TBC) in the combustion chambers [7]. A missing or degraded coating will lead to overheating of the combustion chamber components. In Figure 2 the typical construction of a single-shaft gas turbine is shown. The air is led via the inlet housing in the compressor section and compressed. In the combustion chambers, the air is heated to 1200 °C. In the turbine section, the hot gas expands and leads to highly loaded hot gas components. Part of the energy is needed to drive the compressor; the other part is fed into the electric generator for power generation.

**Figure 3:**
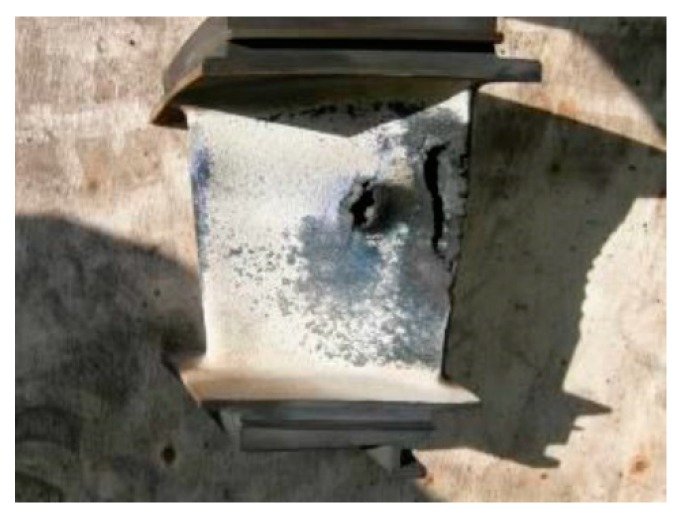
Overheated turbine vane.

**Figure 4:**
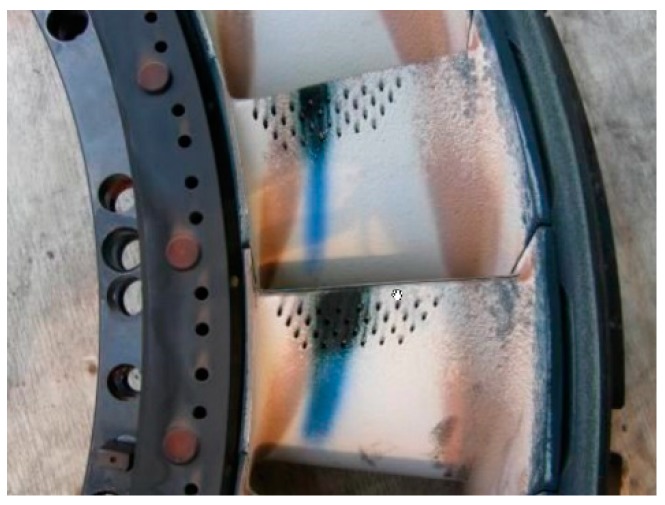
Overheated turbine vane inside machine.

**Figure 5:**
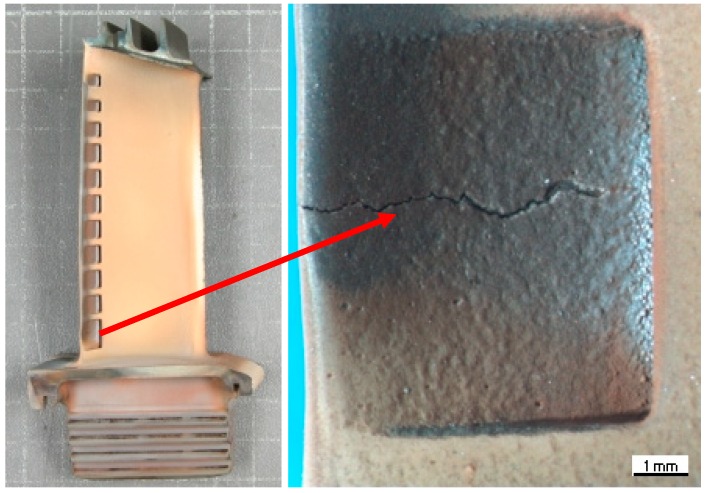
Tear in turbine blade.

**Figure 6:**
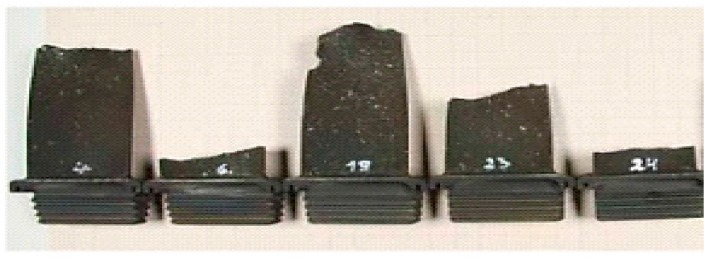
Cracked turbine blades.

The overheated turbine vanes are shown in Figure 3 and Figure 4. In Figure 5, an incipient tear in a turbine blade is illustrated, while in Figure 6, cracked turbine blades are displayed.

Examples of failures in turbine blades in military jet engines with the failures analysis are reported in [10,11,12].

## 3. Hot Gas Component Monitoring

Historically, a common principle to detect vibration and frequencies in rotating blades is with the use of strain gauges. The application of strain gauges is very complex and not every blade can be equipped with these sensors. Furthermore, they are not suitable for long-term operation. Nowadays, several technical alternatives exist with different advantages and disadvantages.

In [13] a historical summary of the use of strain gauges is reported. Kestner et al. studied the correlations between different installed sensor types on blade vibrations. The authors used acoustic pressure, bearing vibration, tip timing and gas path measurement from an operating machine [14]. 

Guo et al. developed a new method to identify engine order, amplitude, natural frequency and damping coefficients of the blades using three casing mounted sensors [15]. 

### 3.1. Tip Timing Method

The tip timing method allows for the possibility of measuring, in-situ, the dynamic behaviour of a turbine blade. The radial clearance between the sensor and blade tip can be measured and also the lateral movement of the blade tip. Additional information like time of arrival can be evaluated (Equation (1)), which gives information about blade vibrations:

(1) $$d_{i}=\frac{2\pi r}{T}\cdot ∆t_{i}$$

where $d_{i}$ is the deflection of blade *i*, *r* is the turbine radius, *T* is the period for one revolution and $∆t_{i}$ is the difference between theoretical and practical time of arrival for blade *i*.

**Figure 7:**
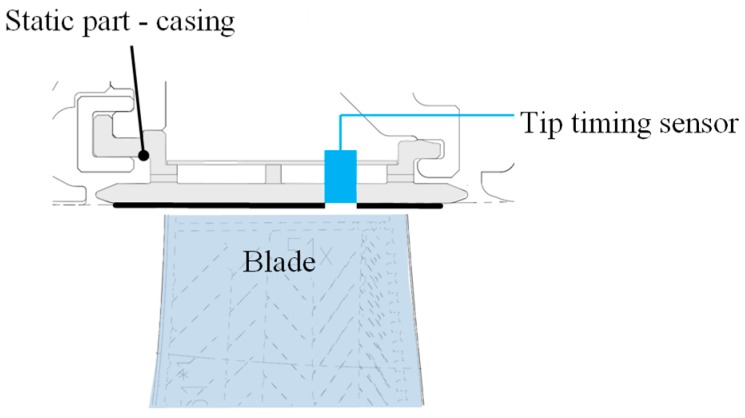
Design of a tip timing system.

In Figure 7 the design of a typical tip timing system is outlined. 

One general problem is that the circumference clearances could differ because of the ovalisation of the casing [16]. 

**Figure 8:**
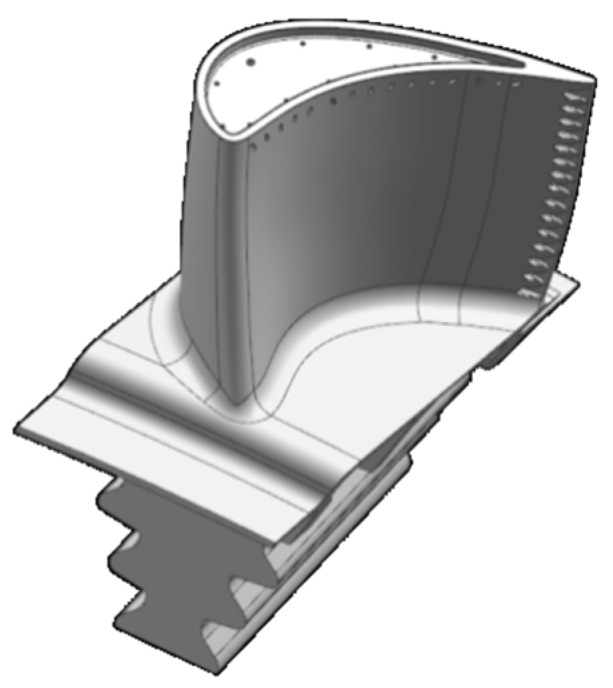
Turbine blade.

A significant challenge is the small changes in position of the blades during an engine starting and stopping. This is because a small clearance exists between the fir-tree of the turbine blade and the turbine disc. Therefore, the mechanical repeatability is not high [16]. The blade tips of gas turbine blades, often have a complex geometry, which could be problematic for failure detection with some tip timing sensors [17,18]. Figure 8 shows the tip geometry of a typical turbine blade.

With the tip timing monitoring technologies, the following failure modes could be detected:

- Crack detection
- Creep deflection
- Rubbing/wear

Witos et al. showed a theoretical approach for health monitoring of compressor blades. With the tip timing method they tried to answer why a blade cracked and with the metal magnetic memory method the localisation of the crack position was determined [19,20]. Rokicki et al. analysed bearing failures of a jet engine with tip timing. Experiments with damaged and working bearings were compared [21]. Tamura et al. focused on non-contact vibration measurement in gas turbines and different sensor technologies were used [22]. Dimitriadis et al. developed a mathematical model to simulate blade tip timing tests [23]. Gallego-Garrido et al. compared different analysis methods for tip timing data [24,25]. The uncertainties of blade tip timing were studied by Russhard [26] and Satish et al. [27].

#### 3.1.1. Microwave Probes

**Figure 9:**
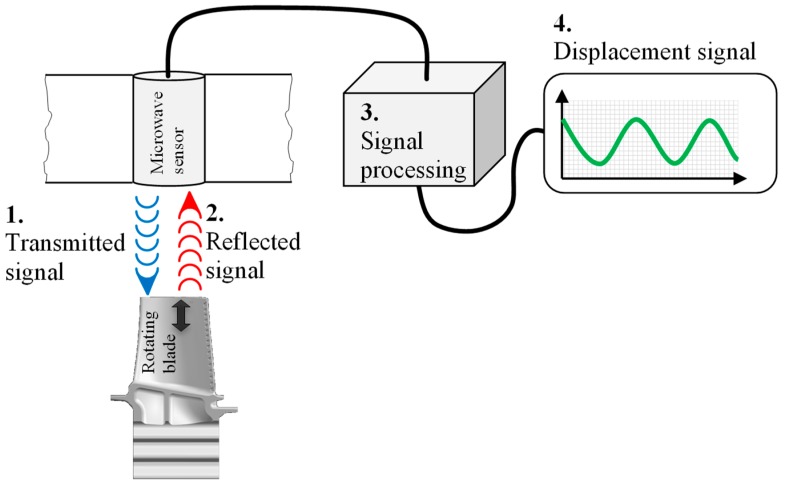
Microwave tip timing system.

Microwaves can be used to measure tip clearance. Microwaves are electromagnetic waves that propagate in the GHz range. A typical microwave tip clearance probe includes the transmittance and a receiving antenna. The microwave sensor sends a continuous microwave signal to the target and measures the reflected signal. This signal is then compared with an existing reference signal and the phase difference with the distance to the blade. The phase differences of the reflected signal are directly proportional to the distance between the sensor and the target [28]. The whole system is shown in Figure 9.

A typical transmitted signal is:

(2) $$X_{0}=A_{s}\cos\left(\omega_{s}t+\varphi_{0}\right)$$

The received signal is:

(3) $$Y=A\left(t\right)\cos\left(\omega_{s}t+\varphi_{0}+\varphi_{l}+\varphi \left(t\right)\right)+A_{r}\cos\left(\omega_{s}t+\varphi_{l}\right)$$

where $\varphi_{l}$ is the summed phase in the transmission path and  φ(t) is the phase difference caused by the change in the tip clearance. The term $A_{r}\cos\left(\omega_{s}t+\varphi_{l}\right)$ is the reflected signal from the sensor itself. This should be removed later in the signal processing [29]. Temperature factors of phase $\varphi_{l}$ can be obtained by setting the frequency outside the sensor bandwidth. Thus the tip clearance can be determined from φ(t) after the calibration.

The blade damage will be detected by measuring dimensional changes reflected in the blade tip clearance [16]. These sensors have a large bandwidth and are able to operate at high temperatures [29,30]. Practically, it was shown that a temperature of 900 °C is feasible [31,32]. Furthermore, the temperature influence was tested, and high temperature measurements are possible [30]. One challenge is the change of phase with temperature, which can be correlated [16]. A validation with optic tip timing systems showed that microwave tip timing systems achieve good accuracy [16,29]. Some failure can develop over a long period of time [29], so accurate sensors are essential.

Repeatability errors are caused by [16]:

- Drifts in electronic component
- Sensor material (change due to temperature effect)
- Contamination of the probe

Compared to eddy current (EC) probes, microwave sensors have a superior resolution and measurement range [30]. Typically the sensors have an outer diameter between 8.5 and 10 mm [17,31]. Many types of damage show just a small change in tip clearance, for example, blade root cracking is around 0.025–0.1 mm (small crack). A sensor for this application needs a sensitivity of at least 0.05 mm, and test conducted showed sensor sensitivities of 0.025 mm [16]. Violetti et al. showed that these sensors are suitable for long-term use [30].

Microwave sensors are a technology that requires further investigation. In particular, the calibration techniques are not fully developed [33]. The distortion of asymmetric turbine casing can be caught with more probes. Typical 4–8 sensors are used [16]. 

The first application in a gas turbine was reported by Wagner et al. in 1998. For pilot tests a 65 MW gas turbine were used [34]. Zhang et al. proved experimentally the general feasibility of blade tip timing with microwave sensors [29]. Woike et al. especially focused on the turbine blades. 5.8 GHz and 24 GHz sensors were used, to measure successfully the blade tip clearance [33]. Hafner et al. investigated the possibility for online tip clearance measurement with microwave sensors and the immediate visualisation of the results [35]. In field machines no applications of microwave sensors were found. For SHM, this monitoring technology is very promising. 

#### 3.1.2. Optical Sensors

A laser Doppler vibrometer (LDV) is a measuring device to determine the vibration frequency and vibration amplitude where, the laser is focused on the surface to be measured. Due to the doppler effect, the frequency of the backscattered laser light shifts as the surface to be measured moves.

If the wavelength of the light is superimposed on that of the vibrating object, the following result is obtained for a 180° scattering angle:

(4) $$∆f_{D}=f_{L}-f_{O}$$

(5) $$∆f_{D}=\frac{2V}{\lambda }$$

where $∆f_{D}$ is the resulting frequency, $f_{L}$ is the frequency of the light, $f_{O}$ is the frequency and *V* is the velocity of the vibrating object.

**Figure 10:**
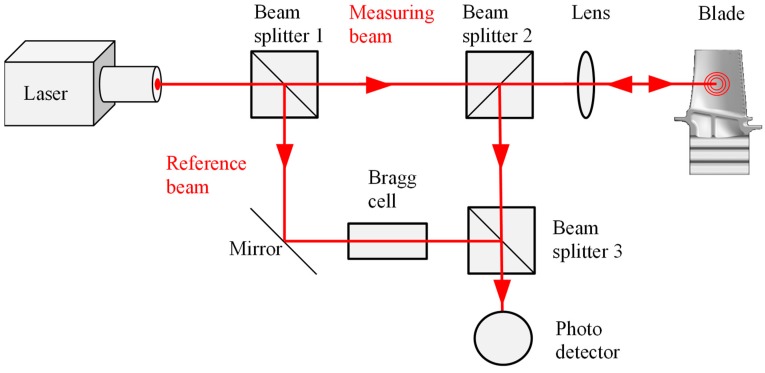
Principle sketch of a laser Doppler vibrometer system.

In Figure 10, the basic structure of an LDV system is outlined. The reference beam does not leave the LDV. It is directed via a bragg cell onto the photodetector, where it interferes with the reflected measuring beam. The bragg cell is an acousto-optic modulator and shifts the frequency of the reference beam. The result is a frequency-modulated voltage, which is directly proportional to the speed of the measured object. Using fast Fourier transform (FFT), the measurement results can be further analysed.

Laser Doppler vibrometers (LDVs) are non-contact vibration measurement instruments. They can also be used for tip timing.

A scanning LDV (SLDV) allows for scanning of whole surfaces. This technology has high sensitivity, even when the sensor is not placed directly on the blade [36]. Optical sensors have good time accuracy and are small sized with a large bandwidth [29,37]. They have an excellent lateral and spatial resolution, a fast response and are often used because of their reliability and repeatability [38]. SLDVs are a good solution to detecting the dynamic shapes of a structure [39]. 

Recent studies have shown that optical fibre probes are suitable for development testing [40]. However, outside the laboratory, debris and low tolerances make their application difficult [17,38,41]. The reported shading problems are disadvantageous [42], as are the optical waveguides with limited operating temperatures and the required inspection window to the blade tip that may be polluted within a short period of time. Therefore, optical sensors are not optimal for long-term instrumentation [37]. To counteract these disadvantages, the use of purge air for cooling was proposed. However, this means additional systems with additional weight. Optical techniques are dominant in the available literature [41]. To monitor the dynamic behaviour of blades in development machines, this is an important technical solution, but for long-term use, it is considered challenging for SHM applications.

Reinhardt et al. made experiments to determine vibration frequencies and amplitudes of turbine blades with a laser vibrometer [43]. Lezhin et al. compared different vibration measurement systems with numerical calculations [44]. The investigation of bladed disks with optical laser probes were publicised by several authors [45,46,47,48,49]. In-situ tip clearance measurements with laser doppler method were presented by Büttner et al. [50,51]. Gil-García et al. [52] and Zielinski et al. [53] focused their work on time of arrival measurements on compressor and turbine blades, to determine blade vibration. Overton developed an LDV system to measure tip clearance more accurately, compared to the capacitive method [54]. Sharma et al. [55] and García et al. [56] used laser vibrometry for SHM aspects. To measure the whole rotor deformation during rotation, Günther et al. made experiments with optical sensors [57]. Oberholster et al. explored a new approach to detect blade damages with Eulerian LDV [58]. Pfister et al. investigated the application to measure simultaneously position and velocity on moving rough surfaces with only one sensor [59]. 

#### 3.1.3. Inductive Sensors 

**Figure 11:**
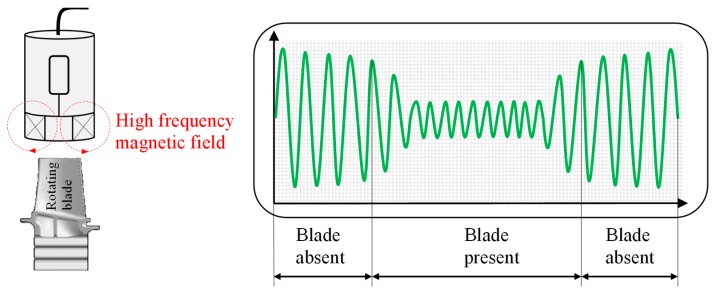
Principle inductive sensor.

Inductive sensors consist of multiple mini-sized planar spiral coils. The sensor measures the tip clearance by measuring the inductance change of planar spiral coils by the passage of the rotor blades. The smaller the tip clearance, the higher the inductance drops, due to the larger EC induced in the blade tip [17]. As the turbine blades passes the magnetic field of the sensor, an eddy current flows because of the electromagnetic induction. This increases the induction current flow, whereby the load of the oscillation circuit increases and the vibration is damped or stopped. The sensor measures this change (Figure 11).

According to Faraday’s law, Equation (6) is given:

(6) $$U_{I}=\overset{}{\underset{c}{\oint }}\left(\vec{v}\times \vec{B}\right)d\vec{l}$$

where $U_{I}$ is the magnitude of the induced voltage, *B* the course of the magnetic induction, *l* the function of coil length and v the resulting speed.

These sensors typically operate at temperatures below 60 °C. Higher temperatures decrease the magnetisation of the permanent magnet and increase the impedance of the winding. They are characterised by long life, high reliability and resistance to contamination [40]. Another advantage is their simple construction. Clearances are tested from 0 to 5 mm with a resolution of 10 μm at a rotation speed of up to 80,000 rpm. Inductive sensors are of simple design, low in cost and easy to install. They have high resolution, high sensitivity and the capability of monitoring a large number of tip clearances simultaneously. These sensors do not need a penetrating hole through the casing. Therefore, the device is more sensitive to the relative vibration between the casing and the sensor. However, this only works if the casing does not contain ferrous material, because the magnetic field, and thus the output signal amplitude is reduced significantly. The sensors cannot detect variation in tip clearances of less than 50 µm. The main disadvantages are the calibration effort and the limited lateral resolution. These sensors can only detect one tip clearance at a specific location at the blade tip. Therefore, for advanced health monitoring techniques, multiple sensors are needed. The low temperature resistance of the sensors does not allow to be used in the turbine part of a gas turbine. However, for the first compressor stages of a gas turbine, their use is still conceivable.

Przysowa et al. developed a new inductive sensor for blade health monitoring systems for military turbofans. Also the resistance to high temperature and contamination were verified [40,60]. Du et al. developed multiplexed inductive sensors for detection of blade tip clearances [17].

#### 3.1.4. Eddy Current Sensors

EC sensors emit a high frequency electromagnetic field. The passing blade in this field induces ECs in the blade tip, acting against the existing high frequency field. The following change in coil impedance can be measured [61]. 

The general expression is shown in Equation (7):

(7) $$V\left(t\right)=n\cdot \frac{d\Phi }{dt}$$

where *V* is the sensor voltage, Φ the magnetic flux passing through the coil and *n* is the number of turns on the coil.

**Figure 12:**
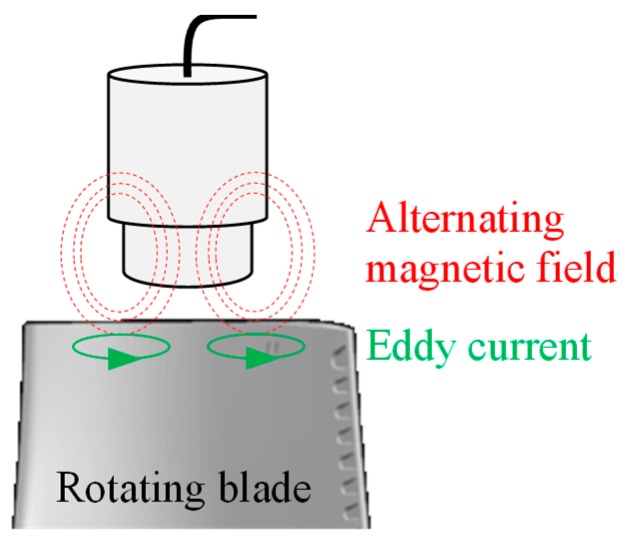
Principle of an EC sensor.

This principle is shown in Figure 12. The determination of the change of this phase position is the main difference compared to the inductive sensors. 

Parts of the sensor start to melt at 93 °C [62]. It was reported that some companies have developed EC sensors that are able to operate at high temperatures up to 1000 °C if an air-cooling system is used [63]. EC sensors have good accuracy [29] and are typically embedded in the casing and the arrival time of the blades will be measured. From this basis, blade deformation can be calculated. They do not work well with non-conductive materials (composites). Furthermore, it is difficult to measure high vibration frequencies without a priori knowledge [64].

Cardwell et al. developed a new EC sensor for blade tip timing of engine fans [65]. Lui et al. proposed a new method to improve the measuring accuracy of EC sensors by considering torsional vibration [66]. Ghana et al. showed new tip timing algorithm for EC sensors which was verified in laboratory and real engines [67]. Tsutomu et al. published their work on the usage of EC sensors for displacement measurements successfully [68]. Przysowa et al. studied health monitoring techniques with EC sensors for military aircrafts [69].

#### 3.1.5. Magnetoresistive Sensors 

Magnetoresistivity is the effect that describes the change in the electrical resistance of a material by applying an external magnetic field. These include, in particular, the anisotropic magnetoresistive effect (AMR effect), the giant magnetoresistance effect (GMR effect), the colossal magnetoresistance effect (CMR effect), the tunnel magnetoresistance effect (TMR effect) and the planar Hall effect. The use depends on whether the component to be examined is magnetic.

To describe the strength of the respective magnetoresistive effect, the quotient of resistance change and resistance without external field is used:

(8) $$\frac{∆R}{R\left[\%\right]}=\frac{R\left(H\right)-R\left(0\right)}{R\left(0\right)}\cdot 100$$

where R(H) is the resistance in dependence of the magnetic field, R(0) is the resistance without external magnetic field, $\frac{∆R}{R}$ is the characteristic of the magnetoresistive effect. 

**Figure 13:**
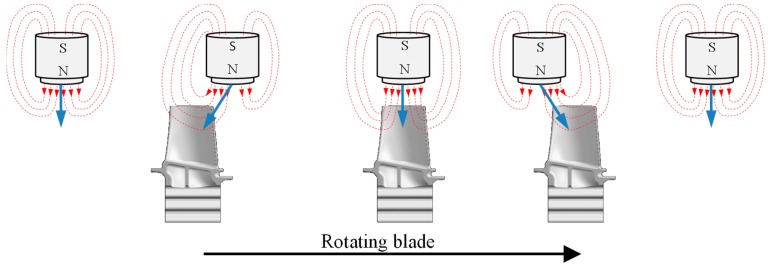
Function of a magnetoresistive sensor.

When the blade passes the sensor, the magnetic field will be distorted. This variation can be measured (Figure 13).

The main advantage of this technology is the relatively small sensor size. Typically, the sensor has a maximum outer diameter of 8 mm. In addition, the simple design lowers the sensor costs [38,42]. They have good tolerances to debris, which is an important for the accuracy and long-term operation. The fast rise time (~20 ns) is also advantageous and their high signal repeatability and time accuracy is comparable to the optical systems and the clearance measurements are in line with capacitive sensors [42].

A new development shows that these sensors have the potential to survive up to 700–800 °C. However, the robustness, durability and accuracy has to be tested [63].

Procházka et al. made fundamental research on this sensor technology in turbomachinery. An online system was developed to monitor the vibrational amplitudes and frequencies of all blades. Possible blade damages could be notified and also blade elongation and blade twisting was shown [70,71]. Brouckaert et al. developed a new magnetoresistive sensor for non-contact blade vibration measurements [72]. Tomassini et al. presented a new sensor design as well [73,74]. The new sensor was successful verified in test benches and in a jet-engine.

#### 3.1.6. Capacitance Sensors

**Figure 14:**
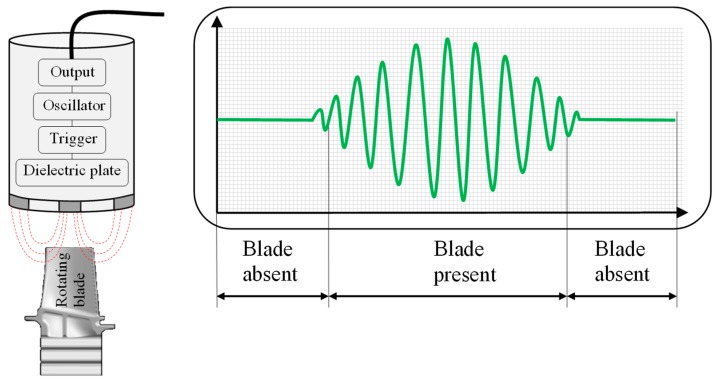
Principle capacitance sensor.

The electrical capacitance, between two electrically conductive materials insulated from one another, is equal to the ratio of the charge quantity *Q* and the electrical voltage *U* ($C=\frac{Q}{U}$). The change in electrical capacitance can be used inter alia to determine distances. In our case capacitance sensors measure the capacitance change between the probe and the blade tip. When the capacitor plate generates an electrostatic field and a turbine blade is present, the capacitance changes so that the oscillator begins to oscillate (Figure 14). Equation (9) shows the relationship of capacitance, sensor geometry and rotor to stator gap:

(9) $$C_{x}=\frac{\varepsilon_{r}\varepsilon_{0}S}{d}$$

where $C_{x}$ is the capacitance from sensor to blade, $\varepsilon_{r}$ and $\varepsilon_{0}$ are the permittivity of the medium and the vacuum, *S* is the area between two sensor plates and *d* is the distance from blade to sensor.

The use of plastics limits the operating temperature of some sensors to 200 °C [75]. Capacitive sensors are predominant due to the improved temperature robustness [37] compared to the other sensor technologies. However, in a gas turbine, the thermal load of the sensors is much higher, therefore, cooling is required. The sensor diameter is relatively small [75] allowing for easier implementation in the engine.

The main disadvantages are the calibration effort and the limited lateral resolution. Some sensors have limitations in bandwidth [29]. The electronic circuits of these systems have to be placed only a few centimetres away from the probe head. The distance from the probe head to the first amplifier has to be at a maximum of 1 m. The output of the probe is nonlinear. For a high precision, the blade tip geometry must be considered. Therefore a "calibration wheel" can be used. With increasing distance between the probe head and blade tip, the signal-to-noise ratio (SNR) decreases. Also, a minimal clearance exists. These sensor types have low costs and are of a simple design [76]. However, the measured capacitance often not only gives the correct clearance because the dielectric property of air could change, due to variations in pressure and humidity [17].

Capacitance probes have a greater potential than optical probes because it is difficult to measure tip clearance with them [41].

Sarma et al. [77] and later Drumm et al. [78] designed a dual-amplifier circuit configuration for tip clearance measurements. Mönch et al. [79] and Müller et al. [80] developed a tip clearance system for compressor and turbine blades. For the usage in micro gas turbines, Fabian et al. described the special requirement of a capacitive tip clearance measurements system in this application [81]. Lavagnoli et al. studied the implementation of a high frequency capacitive sensor on a large transonic turbine stage [82]. These works show that these sensors can be used in hot gas sections for industrial gas turbines for health monitoring of turbine blades.

### 3.2. Vibrational Monitoring

Vibration sensors, like accelerometers, displacement sensors and velocity sensors, are the widest used techniques for blade fault diagnosis in field conditions. 

**Figure 15:**
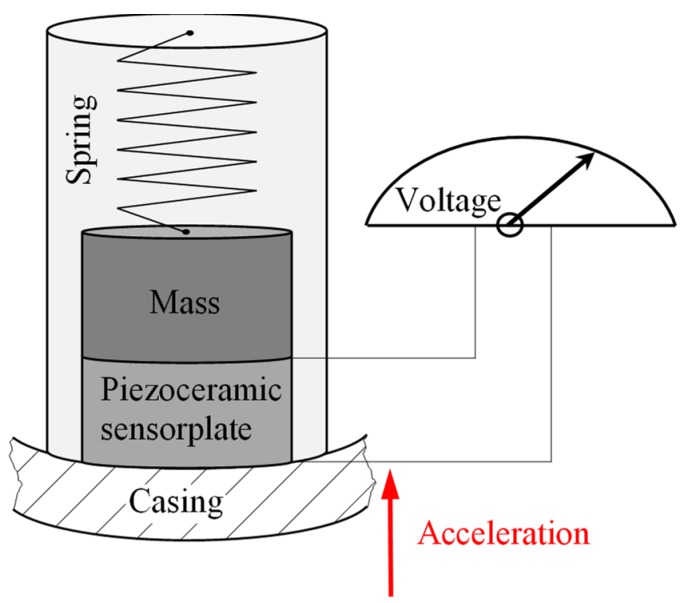
Principle accelerometer.

In industrial gas turbines eddy current proximity transducers are usually used on the bearings to measure the rotor vibrations. The same sensors are also used in the keyphasor to determine the phase angle and the axial position of the rotor [83]. The functional principle is identical to that described in Section 3.1.4. For measuring the casing vibration mostly accelerometers are used. A piezoceramic sensor plate converts dynamic pressure fluctuations into electrical signals. The pressure fluctuation is generated by a seismic mass attached to the piezoceramic and acts on the piezoceramic when the overall system is accelerated (Figure 15).

These signatures combined can be taken for health prognosis of the blades. For the frequency analysis, the most common technique is the frequency spectrum analysis technique. This means the conversion of the vibration signals from time domain into the frequency domain [84]. By analysing these frequencies, the location and failure types can be detected. These sensors work with a frequency of 10 kHz [85]. This method is effective in detecting severe blade faults (e.g., terminal rubbing), while minor faults (e.g., impending rubbing) are mostly not detectable. Therefore, vibrational analysis is not a reliable tool for SHM in field engines [86,87,88]. 

Südmersen et al. showed a combination of pressure measurements, casing vibration measurements and shaft displacement [89]. Lebold et al. presented a work to demonstrate the feasibility of torsional vibration measurements for shaft crack detection. It was mentioned that the same technique may be used for crack detection in turbine blades [90]. In a case study, it was shown that when blade faults are the only failure in the gas turbine, they will often not readily be detected with conventional vibration measurements. The reason is that these failures do not generate enough excitation compared to the other vibration amplitudes in the machine [91]. Alternatively, it was demonstrated that blade related failures could be detected before catastrophic events occur [84]. Ghouti et al. used the shaft torsional vibration signals to extract blade vibration signatures [92].

Zielinski et al. descripted the configuration of different measurement systems with two different probe types [53]. Zhang et al. studied the start-up vibration signatures of an industrial gas turbine for condition monitoring [93]. Sinha et al. presented a study to reduce the number of vibrational probes by improving the signal processing [83]. Loutas et al. tried the combination of vibration, acoustic emission and oil debris for condition monitoring [94]. Schlagwein et al. focused on mistuning effects on blades [95]. In [96,97,98,99,100] it was shown, how condition based maintenance with machine-learning approaches were realised. To detect blade damages with vibrational analysis following papers were published [23,92,101,102,103,104,105,106,107,108,109,110,111,112,113,114,115,116]. Several books were written with focus on vibration of rotating machines [2,117,118,119,120]. 

Despite some disadvantages of this technology, this is a long-proven and cost-effective monitoring technique in gas turbines. This also plays an important role in the performance monitoring (Section 3.11).

### 3.3. Ultrasound

Ultrasound is a common non-destructive testing method for detection of crack or material changes [121,122,123]. Ultrasonic waves usually propagate in a band from 20 kHz to about 1 GHz [124]. 

The ultrasonic harmonic waves presented can be described with the general wave equation:

(10) ψ=Asin(kx−ωt+ϕ)

where ϕ is the initial phase angle, *k* the wavenumber with the wavelength λ=2π/k, the period *T* = 1/*f* and the frequency f = ω/2π. Typically an ultrasonic wave is sent and the reflected signal from the crack can be used for failure prediction. The most commonly used piezoelectric transducers for SHM in gas turbine applications are limited in operating temperature. 

The most relevant guided waves for gas turbine are Rayleigh waves and Lamb waves. Many authors are researching these waves for early crack detection and determination of crack sizes [1,125,126,127,128,129,130]. 

Rayleigh waves are the simplest forms. The longitudinal and shear motions are linked and propagate at a common velocity. The terms “surface acoustic waves (SAW)” and “Rayleigh waves” are usually used as equivalents. 

In addition to the possibilities presented below, surface waves with angled sensors are generated on the component. The disadvantage is that a coupling fluid is needed. Furthermore it should be noted, however, that higher-frequency waves penetrate less in the material than low-frequency waves. For Rayleigh waves, the penetration depth is about one wavelength [131]. These waves are very promising for NDT&E. Especially in high-temperature components, cracks will develop near the surface. Therefore Rayleigh waves are a good tool for inspection [132]. They can be used to evaluate the elastic properties of samples and coatings. The phase velocity of the SAW depends on elastic tensor of the material. This means that SAW information can determine the mechanical properties of components. The phase velocity is sensitive to see small changes in material density, Young’s modulus and Poisson’s ratio [133].

In a far-field characterization, the spatial resolution is half a wavelength (λ/2). Therefore, it is important to use very high frequencies to detect micro-cracks. The disadvantage is that at very high frequencies SNR problems occur. Far-field methods are unsuitable for the detection of micro-cracks. In contrast, in the near-field the spatial resolution is much better [134].

Lamb waves are elastic waves that propagate in solid plates whose particle motion lies in the plane that contains the direction of wave propagation and the plate normal (the direction perpendicular to the plate). Basically deflections occur both in the propagation direction (longitudinal) and vertically (transversal). Lamb waves are dispersive in nature. The speed of propagation *c* depends on the frequency (or wavelength), and elastic constants and density of the material. Therefore, Lamb waves are mixed pressure and shear waves [124]. To determine the relationship between wave velocity and frequency of a sample, dispersion curves can be used. Lamb waves for damage detection are important because they can propagate over long distances and they can show complex phenomena [123,125]. 

Lamb waves consist of the fundamental symmetric mode S_0_ and the fundamental antisymmetric mode A_0_. These modes occur for each excitation frequency. For Lamb waves of short wavelength, several modes of oscillation occur for one wavelength. These are used for symmetrical and antisymmetric Lamb waves (S_0_, S_1_, S_2_ and so on, respectively A_0_, A_1_, A_2_ and so on). These modes have different wavelengths and propagation speeds. They interact differently with small disturbances and mode conversion could occur in presence of of damage or other changes [135].

Linear ultrasonic technology focuses on the measurement of the velocity of sound, attenuation, transmission coefficients and reflection coefficients for crack detection [1,136,137,138].

Nonlinear ultrasound techniques provide the ability to detect creep and thermal aging. Generation of the second harmonic or higher order harmonics can be used for accurate flaw detection. The relationship between the amplitude of the second harmonic wave and the fundamental wave is a proven approach [1,139,140,141,142,143,144,145,146].

Berwig et al. focused on examining the blade root of a turbine blade with Rayleigh waves for surface cracks. These blades are made of γ-titanium aluminide and are brittle. After manufacturing the blade root, this investigation serves the final quality control [147,148,149].

Lane developed an ultrasonic array system to inspect single-crystal turbine blades in-situ [150]. Ultrasonic phased arrays consist of several ultrasound transducers, where the excitation time and amplitude is controlled individually by computer. This gives the possibility, to focus the ultrasonic waves. This leads to a more accurate statement about the crack position and size. In addition, the SNR is improved [151,152]. Chatillon et al. has dealt with complex geometries to detect errors with the phased array transducer [153].

The next subsections show special applications that are already used for the SHM of gas turbine components or that offer promising solutions for future research.

#### 3.3.1. Waveguides 

**Figure 16:**
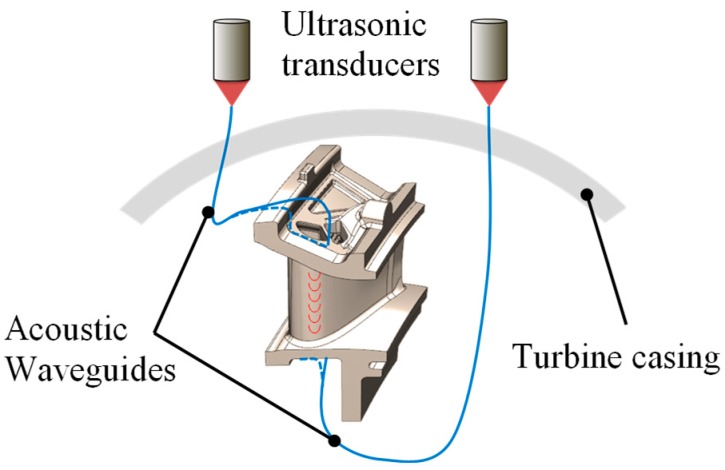
Schematic setup of an acoustic waveguide sensor.

Harold et al. have filed a patent dealing with the condition monitoring of gas turbine components by means of acoustic waveguides (AWG) [154]. The general idea is to send an acoustic wave through waveguides to each vane. The sound waves pass through the vanes and are received by a second acoustic waveguide. A filter removes the lower frequencies below 30 kHz. As the coating on the blade deteriorates the size and/or velocity of the resulting acoustic wave changes. This is an indication that a blade must be serviced. The wave velocity for an intact coating is about 2500 m/s and for a completely deteriorated coating almost 5000 m/s. The principle of this idea is shown in Figure 16. 

The patent also describes the possibility of performing measurements without an acoustic input signal. As the rotating blades spin past the vanes, the blade produces a pulsating gas pressure, causing acoustic waves in the vane. This signal can be measured via an acoustical waveguide.

The inventors gave a good description of how the acoustic waveguides can be designed and fastened with the sensors and vanes. The AWGs are connected to the vanes by either a point or a few inches long. When entering the AWG, the waves are converted into longitudinal waves. They must be a good transmitter for high-frequency acoustic signals (20 to 500 kHz) and be weldable. A variety of metals and composites are also possible. For the investigations of this patent, a wire with a diameter of 1 mm of platinum and platinum/13% rhodium was chosen. They must survive in the environment of hot oxidative gases and high dynamic loads. Typically AWGs use small wires or rods with a diameter of 0.25 to 6.4 mm [155].

Willsch et al. have implemented this concept practically in an industrial gas turbine [156]. Atkinson et al. have studied how the acoustic waves from the transducer are directed into the AWG via a conical transformer [157]. This is an elegant solution to the temperature issue but it is not feasible for industrial applications.

Waveguides are also used for temperature measurement with ultrasound (ultrasound thermometry). The basic idea is to run ultrasonic waves through the waveguide. By increasing the temperature, the waveguide expands. This can be measured in the delayed ultrasonic signal and give conclusions about the temperature to be measured [158,159,160,161,162].

#### 3.3.2. Thermosonic or Ultrasonic Stimulated Thermography

**Figure 17:**
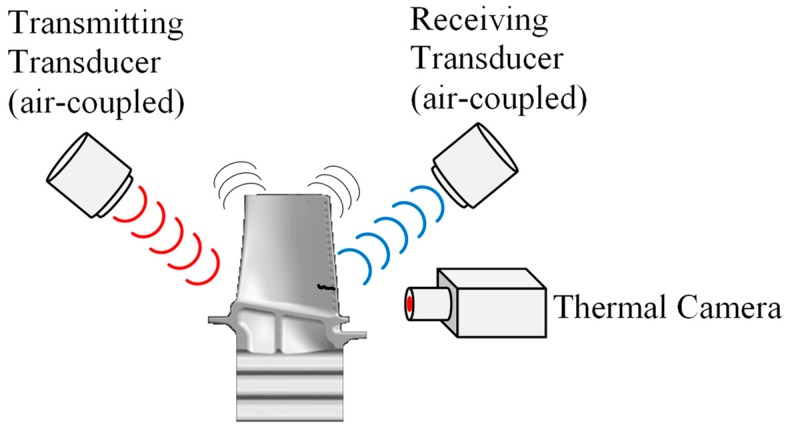
Thermosonic measurements with air-coupled transducer on a turbine blade.

This is a NDT&E method to detect micro-cracks on the surface and sub-surface of test parts. The test object is vibrated ultrasonically with or without contact or air-coupled transducers. As a result, the contact surfaces of the crack rub against each other and generate heat. With an infrared camera, the crack can then be detected (Figure 17). 

Dyrwal et al. have demonstrated that with this technique micro-cracks can be detected on the outer shroud of a turbine blade. In this work different techniques were compared. The non-contact nonlinear air-coupled thermosonics (NACT) technique was the most promising [163]. Zhang investigated the physical process of the chaotic excitation of a turbine blade to improve the fault detection capability of thermosonic technology [164].

Further literature is available for the air-coupled transducers [165,166,167].

#### 3.3.3. Laser Ultrasonic 

**Figure 18:**
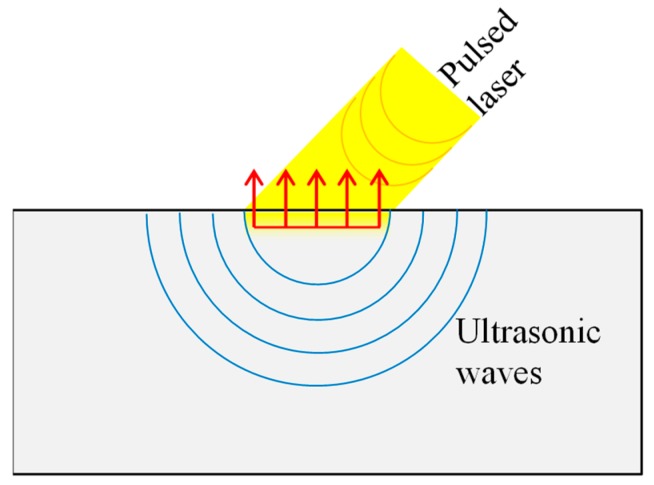
Principle laser ultrasonic.

In laser ultrasound, the ultrasonic wave is transmitted with light. The light of a laser hits a material surface where it is absorbed in a layer near the surface, resulting in heating, thermal expansion and finally emission of an ultrasonic wave (Figure 18). The generated ultrasonic waves propagate perpendicular to the sample surface and are independent of the angle of the laser [168].

Compared to conventional piezoelectric transducers (PZT), ultrasound generation by laser irradiation offers several advantages. No sensor contact is required; they have high spatial resolution and can work on complex designed surfaces. The acoustic waves can be received with PZT or electromagnetic acoustic transducers (EMAT). Laser interferometers on the receiving side are also possible. 

The surface of the component to be examined is not damaged. One disadvantage is the poor SNR with laser ultrasound [169,170]. Masserey et al. and An et al. worked on crack detection using laser ultrasound with focus on complex designed geometries [132,171]. Pei et al. studied the inspection of inner cracks [172]. Dhital et al. investigated a similar topic, but used air-coupled transducers for damage detection [173].

Direct applications of this technology in the turbomachinery sector have not been identified, but this is a promising NDT&E approach. An SHM application is difficult here because laser ultrasonic has the same disadvantages as the optical technique shown above.

#### 3.3.4. High Temperature Transducers

To solve the problem of extremely high temperatures in gas turbines, there are also promising approaches to use high-temperature ultrasonic sensors. On the one hand, it is important to solve the problem that the sensors themselves survive the high temperature, but on the other hand, there must also be a functioning acoustic coupling between the sensor and the test object. La_2_Ti_2_O_7_ (lanthanum titanate) or LiNbO_3_ (lithium niobate) enables operation at higher temperatures (Curie temperature higher than 1000 °C) [63]. Other promising materials are also possible. Many of these ideas are still in developmental status and commercially difficult to make available, but as an SHM approach, shows potential good performance [174,175,176].

#### 3.3.5. EMAT

**Figure 19:**
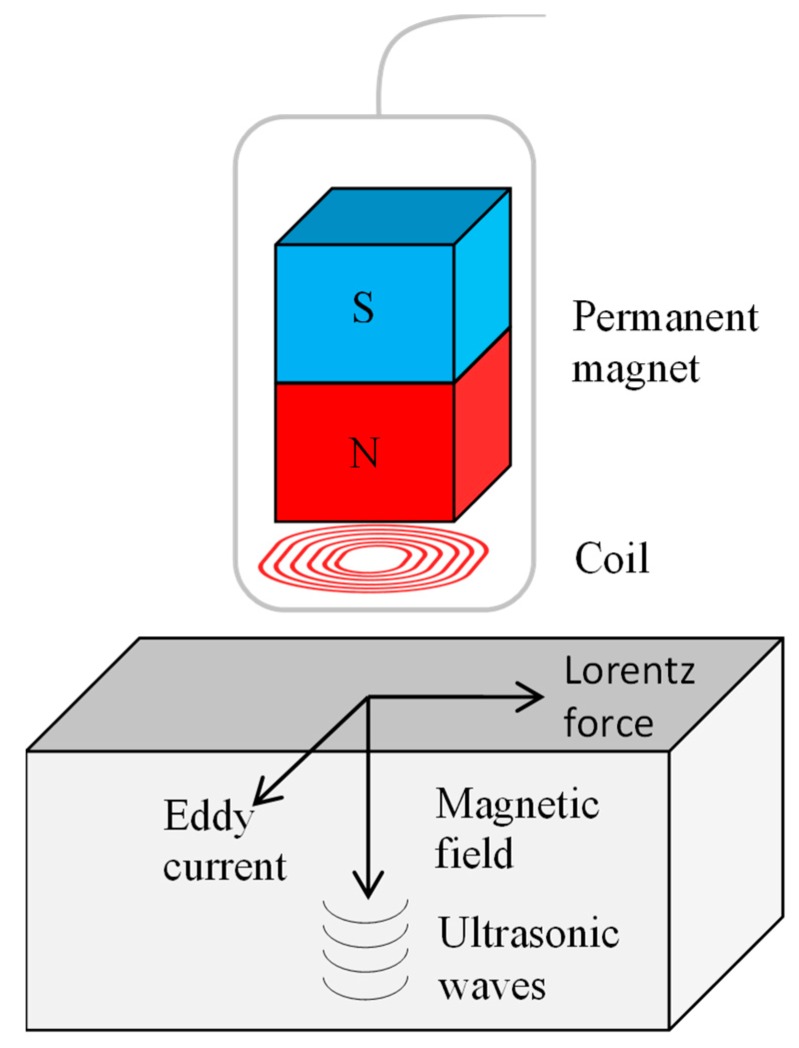
Working principle EMAT.

Alternating current is conducted into the induction coil of the sensor. This generates electromagnetic oscillations, which in turn induce eddy currents on the surface of the test object. The eddy current interacts with the permanent magnetic field of the sensor and generates ultrasonic waves through the Lorentz force directly on the surface of the test object (Figure 19).

The main advantage is certainly that EMATs works contactless and the sensor is located a few millimetres from the test object. That means that no coupling is needed. In addition, a wide variety of ultrasonic wave types can be generated that would not be possible with PZTs. For example, transverse shear waves (SH) can be transmitted and received. They can also be used at significantly higher temperatures. The disadvantage is that metal particles can be attracted by the magnet. In addition, there is the danger of magnetization of the test object. These sensors are typically quite bulky and are not commonly used in aerospace applications [177].

Edwards et al. and Jian et al. researched Rayleigh waves generated by EMAT for crack detection [178,179,180]. Dixon et al. studied a hybrid method in order to bring the ultrasonic waves into the component by means of a laser and to detect them using EMAT [181].

### 3.4. Temperature Measurements

Pyrometry and infrared thermography are good methods to detect creep, low-cycle fatigue, corrosion, erosion and oxidation [87,182]. Phosphor thermometry is able to identify failures such as cracks, erosion, corrosion and wear [183]. These techniques will be presented in detail in the next chapters.

#### 3.4.1. Pyrometry

**Figure 20:**
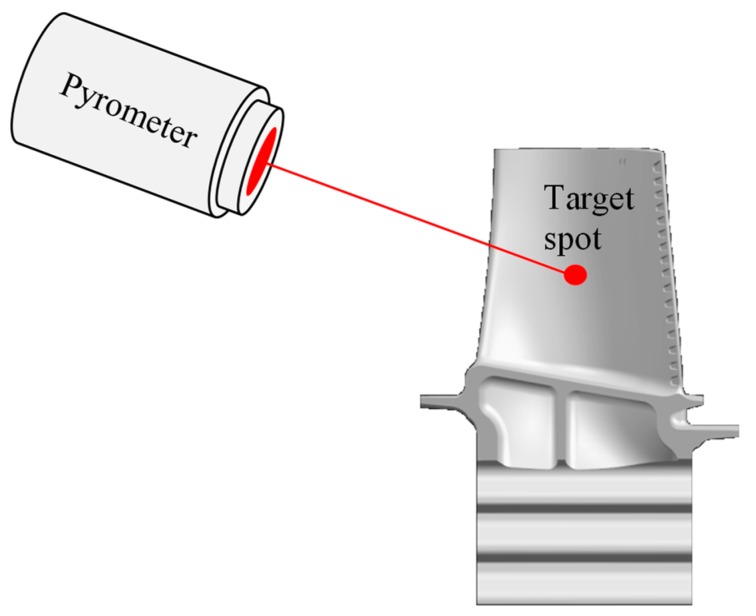
Principle pyrometry.

With this technique, thermal radiation at the target point of the turbine blades can be measured. They are used for non-contact temperature measurement. The emitted thermal radiation of a body can be measured with the help of a pyrometer (Figure 20). 

The relationship between the surface temperature and the emitted radiant energy is defined by Planck’s law (Equation (11)):

(11) $$E_{b}\left(\lambda ,T\right)=\frac{c_{1}\lambda^{-5}}{\exp\left(\frac{c_{2}}{\lambda T}\right)-1}$$

where $E_{b}$ is the monochromatic radiation, *T* the temperature, λ the wavelength, $c_{1}$ and $c_{2}$ the radiation constants.

Pyrometers have the capability to measure ~40 points per blade [184]. To make these sensors work, no upper temperature limit exists. However, a minimum temperature limit of ~500 °C is required. They have a fast response and no physical contact with the turbine blades. Pyrometry is also immune to electromagnetic interference. However, finding a position to install the probe where it can view the blade surfaces is problematic and the optic has to be protected from deposits. The instrumentation is vulnerable to extreme temperatures, vibrations and high pressures. The combustion gases are turbulent with variable densities and high velocities. The sensors in the flow channel have to operate at an oxidising atmosphere. Corrosion is also a failure mode. It could be difficult to gain enough radiation of the small target area of the blades. In addition, records for representative temperature measurements are needed. Difficulties are reported in specifying the emission accurately [182].

The development of a blade temperature management system with pyrometers was reported by different authors [185,186,187,188,189]. Alaruri et al. [190] and Gao et al. [191] analysed the emissivity of different superalloys with TBC and verified the theoretical approach experimentally. Kerr et al. [192] and Daniel et al. [193] presented an overview of measurement and reflection errors associated with pyrometers. 

#### 3.4.2. Infrared Thermography

**Figure 21:**
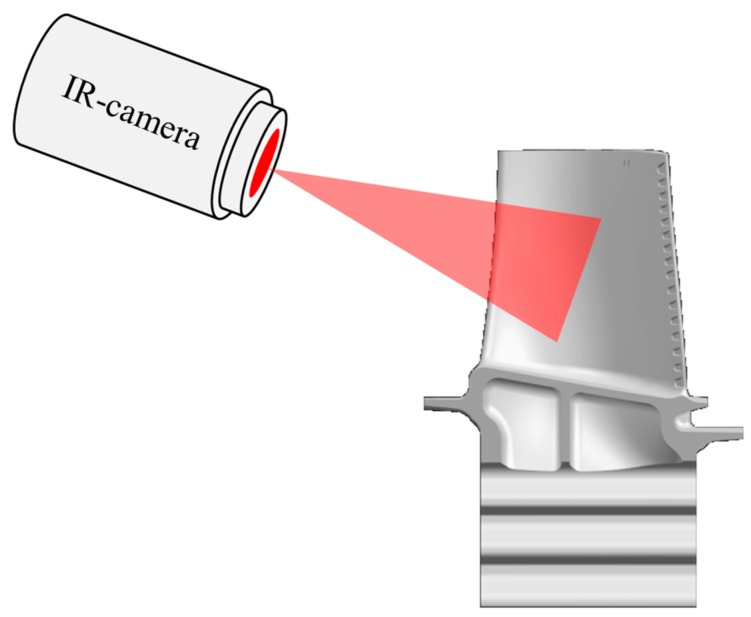
Principle infrared thermography.

This is an imaging process for displaying the surface temperature of objects. Every body with a temperature above the absolute zero emits heat radiation. An infrared camera converts the infrared radiation, which is invisible to the human eye, into electrical signals. From this, the camera creates a picture. Infrared cameras are used to take infrared pictures of the whole aerofoil that has to be evaluated (Figure 21). 

Lemieux from Siemens Westinghouse Power Corporation showed a conceptual design of this technology in gas turbines where two IR cameras were used. These cameras also have a blade positioning sensor allowing tracking of all blades [194]. The automatic comparison of the IR pictures showed temperature increases at specific points of the blade. This could also be used to monitor degradation of the TBC or cracks. 

Bison et al. evaluated especially this topic. They compared pulsed thermography and thermal wave interferometry to estimate ageing effects of the TBC [195,196]. Sun et al. showed a multilayer analysis method to detect change in TBC properties as well [197]. Meola et al. developed a non-destructive testing method to detect small remaining ceramic fragments in the casted core [198]. 

#### 3.4.3. Phosphor Thermometry

**Figure 22:**
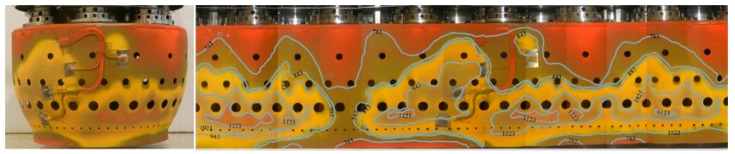
Thermal paint gradients of the combustor outer liner [199], with permission from Begell House, Inc.

The basis of this technique is a thermal history coating. Active rare earth ions are introduced. This could be integrated in the TBC, which is what the hot gas parts in a gas turbine are mostly coated with. By illuminating with an excitation light after a machine run, phosphorescence gives information about the temperature that the coatings experience. Figure 22 shows a combustion chamber outlet liner, with thermal paints, after service.

A temperature capability of −5 to 1550 °C is given and a precision of ±5 °C was proved. The disadvantage of pyrometry and infrared thermography is the lower susceptibility to background radiation. Feist et al. showed in different publications the industrial application of phosphor thermometry. To develop these special paintings a Rolls-Royce jet engine was used for verification [183,200,201,202,203,204]. The application in an industrial gas turbine was proven as well. The surface temperature of combustion chamber transition ducts, turbine blades and sideplates were measured and used for validation for the CFD calculations [205,206]. 

### 3.5. Induction Thermography

**Figure 23:**
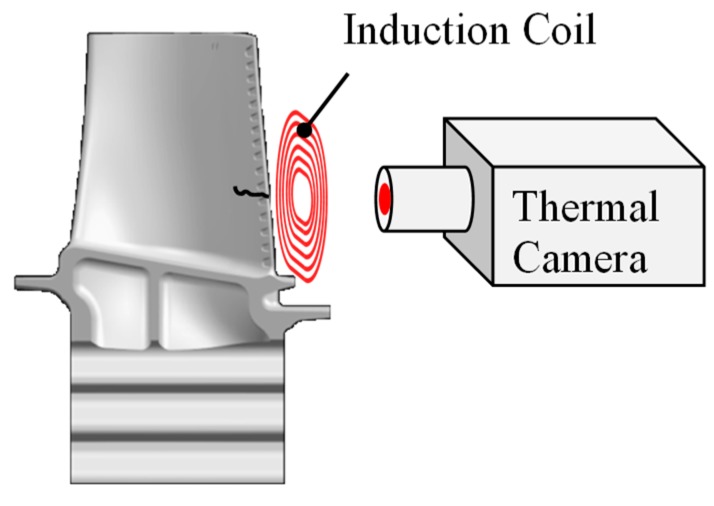
Principle inductive thermography.

In inductively excited thermography, an electrically conductive component is inductively impressed by a near-surface eddy current. The resulting heating of the component can be visualized with an infrared camera (Figure 23). Cracks disturb the current flow and thus also influence the temperature development in the test part. This technique is similar (Section 3.3.2) to thermosonic technique, where the excitation is achieved using ultrasound excitation.

The penetration depth of the electromagnetic field is described by the skin effect:

(12) $$\delta =\sqrt{\frac{2\rho }{\omega \mu }}$$

where δ describes the skin depth, ρ is the conductor resistivity, ω the angular frequency and μ the absolute permittivity. The choice of the excitation frequency determines, inter alia, the depth to be examined into the component. In addition, coil geometry and power are also important test parameters [207].

The method is of particular interest where the crack detection with conventional methods is difficult or impossible. Compared to other excitation techniques, inductive excitation offers the advantage of being less sensitive to radiation or emission differences on a test surface, as the heat is generated directly in the test part [207].

Sensitivity to crack detection on components can be compared to magnetic particle inspection [208]. The advantage of induction thermography compared to magnetic particle testing is that it is non-contact. In addition, the use of chemicals is avoided. Accuracy is limited when components with highly reflective surfaces are tested, which have a low emissivity [208]. 

Carl et al. developed a system for automatic crack detection of turbine blades. A robotic arm puts the blades in test position. After the measurement was carried out, the automatic evaluation of the blades takes place [207]. Šrajbr et al. used induction thermography for automated crack inspection of aircraft structures [209,210]. Bamberg et al. also introduces a system for inspecting turbomachinery components for cracks using induction thermography [211]. Vrana dealt with active thermography and examined cracks in the foot area of gas turbine blades [212].

Spießberger et al. compared inductive thermography with ultrasonically excited thermography [213]. The detectability of cracks in induction thermography depends on their orientation in the component and in ultrasound-excited thermography, this is largely independent. With ultrasonic excitation, the detection of defects is possible at a greater depth than in the inductively excited thermography, since the heat of fracture generated by ultrasound diffuses from greater depths to the surface. With complex geometries, it is difficult to excite the component surface evenly with induction. It is then often only possible to examine individual component areas. If the entire component is to be tested, the use of ultrasound-excited thermography appears to be more sensitive. For materials with low thermal conductivity, ultrasound-excited thermography is more suitable as a test method [213].

### 3.6. Acoustic Emission (AE)

The basis of AE are transient elastic waves. They are generated due to the release of energy in a material when a crack occurs. The frequency range is between 100 KHz and 1 MHz. 

**Figure 24:**
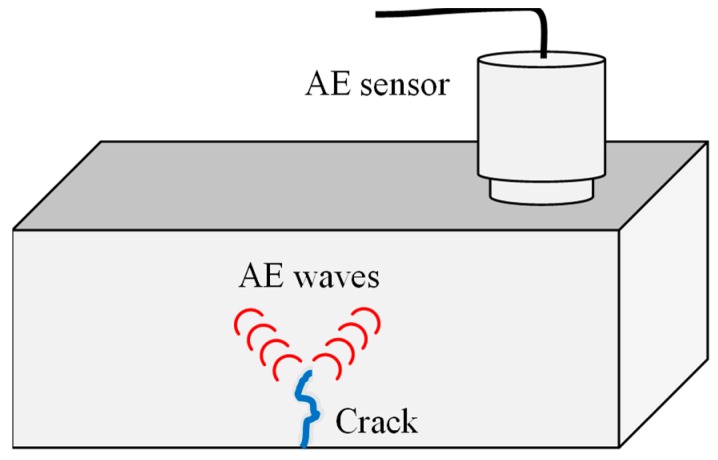
Principle AE measurement.

Defects in a machine generate an individual type of crack growth. This phenomenon results in elastic waves, which can be detected with AE sensors (Figure 24) [85].

Al-Obaidi et al. provided a good explanation on how AE works: “Instead of supplying energy to the object under examination, AE simply listens for the energy released by the object” [214]. 

AE is sensitive enough to monitor minor changes in the gas turbine parts. It has the potential to detect failures at an early stage. Furthermore, the crack position can be detected without intrusion. AE sensors were used to observe roller bearings in gas turbines [215]. It was found that AE is a proper tool to detect rubbing from rotor to stator. 

Mba et al. showed promising experiments for early rub detection in turbine rotors [216]. AE is very sensitive to incipient cracks compared to vibrational measurements. It was shown that the AE technique is able to detect cracks in blades earlier than vibrational analysis [87]. One drawback is the attenuation of the signal. Therefore, the sensor has to be placed close to the signal source [217]. The accuracy of AE is limited because the propagation of the velocity of the acoustic wave depends on temperature, pressure and the relative speed of the medium [37].

Mba et al. developed a AE technique for monitoring rotating machines as well [217]. The focus was on bearings, pumps, gearboxes, engines and rotating structures. Leahy et al. investigated experimentally rubbing detection between rotor and stator of a steam turbine [218,219]. Nashed et al. explored the sources of fluid generated AE in a running turbine [215]. These findings can be used for blade faults predictions. 

AE is a relatively simple and cost effective monitoring technique for health monitoring of hot gas components. These sensors are limited by the high temperatures in the gas turbine, so that use in the hot gas without active cooling is not possible.

### 3.7. Mm-Waves

Mm-waves are microwaves with wavelengths in the millimetre range between 1 and 10 mm. This corresponds to a frequency band from 30 to 300 GHz. The physical principle is the same as described for tip-timing measurement in Section 3.1.1.

**Figure 25:**
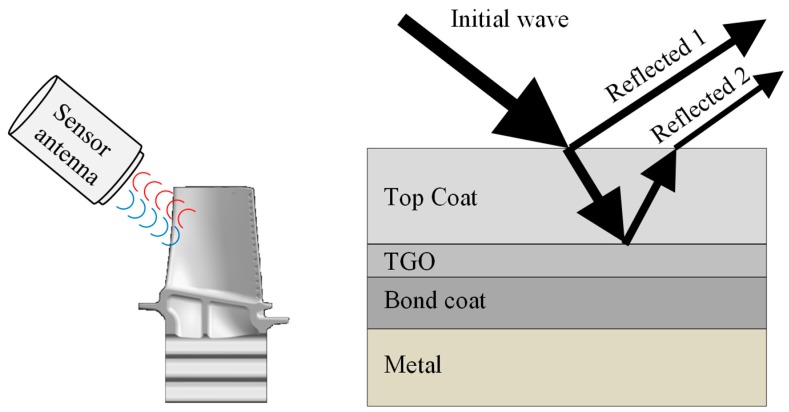
Principle of mm-wave propagation in TBC coating.

The mm-wave technique is commonly used in motion detection and special radar systems. It can be used to measure movements of turbine blades, rotor-stator clearances and the degradation of TBCs. TBCs typically consist of four layers. Starting from the bottom the metal substrate, the metallic bond coat, the thermally grown oxide (TGO) and the ceramic topcoat. Mm-waves are nearly totally reflected by metal surfaces. TBCs have a high dielectric constant of typically 25. The waves will be partly absorbed and differently reflected. These differently reflected waves could be used to detect the degradation of the coating (Figure 25). An antenna sends the waves to the moving blades and the reflected waves will be evaluated. Tests were done with 2.45 and 10 GHz sensors. Further investigations concerning the electromagnetic losses of TBCs are necessary. Willsch et al. have shown this SHM application in a running gas turbine [156].

The degradation of a TBC is a relevant indicator for health monitoring of the hot gas parts. However, an active cooling strategy for the antenna is needed.

### 3.8. Pressure Measurements

In gas turbine casings are placed several pressure sensors. The distortion of the pressure fields around the blades can be used for fault detection [220]. An experimental study was completed to find a correlation between compressor casing vibrations and the pressure field around the compressor blades [87]. The pressure field around the compressor blades gives a clearer picture about the blade fault compared to vibrational analysis [88]. It was shown that pressure measurements deliver better results than common vibrational probes [87]. Mathioudakis et al. found different signatures corresponding to the different blade faults [220,221]. Failure modes, such as blade creep, rotor eccentricity and rubbing, were experimentally detected [88]. Forbes et al. presented a concept to combine internal casing pressure measurements with casing vibration measurements. It was proven that blade defects can be detected [222,223,224,225,226].

### 3.9. Direct-Write Thermal Spray (DWTS) Sensors

**Figure 26:**
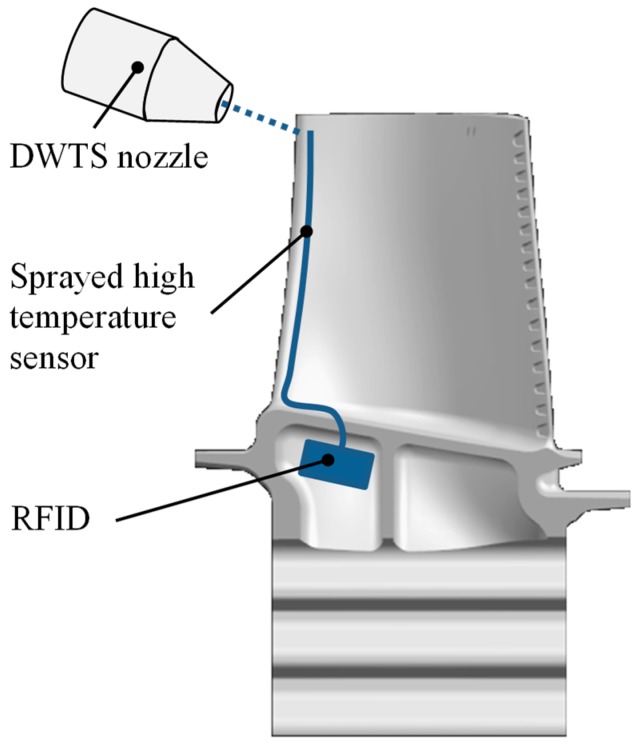
Principle DWTS on a turbine blade.

DWTS is a spray process that accelerates material to high speeds and hits a substrate. This creates a dense and strongly adherent deposit. Typically, the material is injected in the form of a powder, wire or rod into a high velocity combustion or thermal plasma flame. As a result, thermal and kinetic energy is supplied to the particles (Figure 26). This is a similar process to apply TBC to a component [227]. The DWTS process allows the additive generation of sensor circuits on complex shaped components without pre-masking.

**Figure 27:**
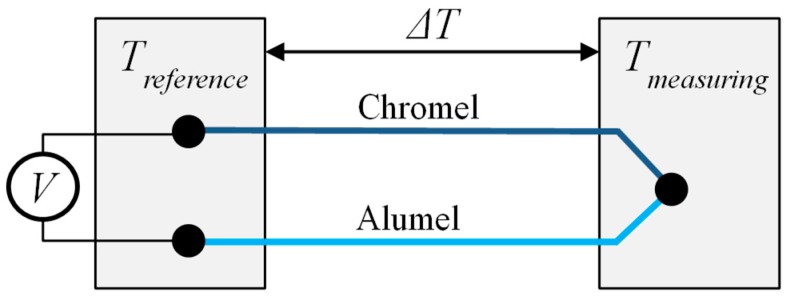
K-type thermocouple.

Longtin et al. have studied the use of temperature sensors and strain gauges manufactured by DWTS. K-type thermocouples can measure high operating temperatures. Industry standard K-type thermocouples are made of Chromel (90Ni/10Cr) and Alumel (95Ni/3Mn/2Al/1Si) [227]. A K-type thermocouple consists of a pair of metallic conductors made of these different metals. These are connected at one end and are suitable for temperature measurement due to the thermoelectric effect. In principle, the thermocouple provides electrical energy from heat at a temperature difference along the electrical conductor (Figure 27).

Schönberg et al. has also applied K-type thermocouples with DWTS direct to the surface of a test component. In this way, in-situ temperature measurement data of the turbine blades can be determined [228]. Zhang et al. has developed a system to weld K-type thermocouples directly onto the TBC, which then embeds the sensor through a second ceramic layer [229].

**Figure 28:**
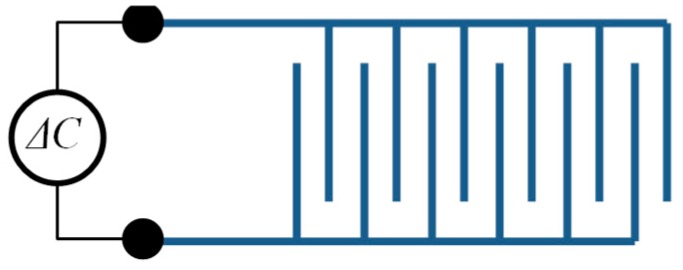
Capacitive strain gauge.

Capacitive strain gauges are well suited for determining the mechanical stress of a component in extreme environmental conditions. Compared to resistive stain gauges, they have a better SNR and are not so sensitive to high temperature and temperature fluctuations. If the fingers of the sensor are displaced relative to each other by the component load, this results in a capacitive change *ΔC*. This is approximately proportional to the strain *ε* [230] (Figure 28).

Li et al. used DWTS and ultrafast laser micromachining to produce the sensor. After spraying a layer, the laser worked out the exact sensor contour [230].

It is advantageous that thermal spray is already used as a standard method for applying a protective layer in gas turbine components. As a result, the sprayed-on layer adheres directly to the component. No adhesive or mechanical connection is necessary. It is also possible to form a capacitor-inductor circuit (LC). This would provide the opportunity to transmit the measurement data passively and wirelessly [230,231,232]. 

Chen et al. dealt with micromachining strain gauges using DWTS and precision laser [233]. Hon et al. have published an overview of the various direct write technologies and their state of development [234]. Pique reports different areas of application in his book on direct-writing technologies [235].

This technique is a promising way to determine the condition of the turbine blades in-situ and may be considered in future research. It will certainly be difficult, due to the high temperature, to use the electronic components for data transmission as shown in Figure 26. Passive data transmission systems would be a good solution.

### 3.10. Uniform Crystal Temperature Sensor (UCTS)

These sensors are particularly suitable for prototype machines to determine the temperature at certain points of the test subject. The crystal sensor is inserted into the test object and a pocket with a diameter of 0.75 mm and 0.75 mm deep must be removed. After inserting the crystal, it is closed again. When the test run is finished, the sensor must be removed for evaluation [236]. The temperature changes the lattice structure of the crystal, allowing an accuracy of ±3.3 °C [237]. The application temperature is 150-1430 °C [237]. DeVoe et al. have studied their use in gas turbine blades [238,239].

### 3.11. Performance Monitoring

Performance monitoring processes different input data. The temperature, pressure and speed of the gas turbine are used to calculate the performance of the machine. This method is also able to detect blade faults. This fault changes the aerodynamic behaviour of the blades, and ultimately, the performance of the whole machine. A combination of performance and vibration monitoring is known as a hybrid method. It was shown that blade deformation, blade wearing and blade fouling could be detected [240]. 

Lattime et al. discussed the degradation of the high pressure turbine (HPT) performance. Especially, wear on blade tip and sealing can account for losses in the HPT performance of 1% and more [241]. Salar et al. developed a method to detect faulty components inside a gas turbine. Therefore, a gas path analysis and extended Kalman filters were used. The main parameters to detect the degradation are efficiency and flow capacity of the compressor and the turbine [242]. Diallo et al. established a new statistical signal processing technique for performance monitoring [243]. A physics-based modelling approach for performance deterioration were shown by Hanachi et al. [244]. Heat loss index and power deficit index were used as indicators. Different methodologies to improve the robustness of performance monitoring against sensor faults were developed [245,246]. Tahan et al. reviewed current gas path performance monitoring techniques. The different failure modes in a gas turbine and the possibilities to detect them with performance monitoring were listed [247]. 

This technique is very promising for future condition based maintenance concepts. Many sensor technologies are already available as standard monitoring in series machines that can be used for the evaluation. A research focus should be on the further development of modern signal processing techniques.

## 4. Conclusions

The most relevant failure modes for gas turbine hot gas components have been described. For blade health monitoring, TMF, creep, oxidation and corrosion are the main life limiters depending on the turbine configuration. For the transition ducts of the combustion chambers, wear of the mating surfaces and degradation of TBCs and cracks are problematic. 

A review of most relevant NDT/SHM techniques for gas turbine was reported. Tip timing technology allows for measurement of the tip clearance of the blades, time of arrival and also axial deflection. This is a very powerful method for which several sensor technologies could be used. Optical measurements are the best choice for prototype machines because of their accuracy and resolution, but the sensitive optics could be problematic for long-term operation in field machines. Inductive and magnetoresistive sensors are not able to work in the first turbine stage because of their limited temperature. Microwave, EC and capacitive sensors seem to be a good choice for turbine tip timing.

The pyrometry enables the measurement of heat radiation at different points of the blades. IR cameras take infrared pictures of every blade during every rotation. Both monitoring techniques compare previous measurements and pictures. Phosphor thermometry is a new innovative method for temperature detection. However, all of them are rather complex solutions for health monitoring in field machines.

AE is a powerful diagnosis technique. The sensor has to be placed close to the signal source. This is a promising method to detect rubbing; however, limited accuracy and signal attenuation of this technique needs can be a limiting factor.

Millimetre waves are a possibility to detect degradation of TBCs. However, a sensor antenna directly placed in the hot gas disturbs the flow and the sensor has to be actively cooled. 

The detection of pressure differences in the flow channel could also be a good indicator for health monitoring of gas turbine components, especially in combination with other technologies. 

The vibrational approach is the classical technique, where for example accelerometers on the bearings and on the casings provide important information on the machine behavior; however, it is known that blade failures could not be detected readily with conventional vibration measurements. 

The ultrasound methods presented offer promising possibilities in the field of NDT&E and SHM. 

The current review reports some current NDT/SHM solutions, able to mostly monitor the components contactless and the results shows that a combination of monitoring technology would provide valuable information about the performance and integrity of gas turbine components.

A summary of the different techniques with the corresponding properties is shown qualitatively in Table 2. Here it is presented which failure modes can be detected with the individual techniques and whether they are primarily suitable for NDT or SHM. In addition, important properties and also the costs are summarized.

## Figures and Tables

**Table 1:** Typical failure modes for hot gas components inside a gas turbine.

Continuous Duty	Cyclic Duty
Rupture	TMF
Creep deflection	High-cycle fatigue
High-cycle fatigue	Rubbing/wear
Oxidation	Foreign object damage
Erosion	Combined failure mechanism (creep/fatigue, corrosion/fatigue, oxidation/erosion and so on)
Corrosion	
Rubbing/wear	
Foreign object damage	
Combined failure mechanism (creep/fatigue, corrosion/fatigue, oxidation/erosion and so on)	

**Table 2:** Summary–Properties of sensor technologies.

	Sensing Technology	Failure Modes	NDT	SHM	Temp. limit	Band-width	Accuracy	Sensitivity	Costs
Tip timing method	Microwave probes	Creep Crack Rubbing Wear		X	+	+	+	0	0
	Optical sensor			X	−	+	+	+	−
	Inductive sensors			X	−	+	+	+	+
	EC sensors			X	−	0	+	0	0
	Magnetoresistive sensors			X	+	+	+	+	+
	Capacitance sensors			X	+	0	0	0	+
	Vibrational monitoring	Rubbing FOD Crack		X	0	+	-	-	+
Ultrasound	Waveguides	Crack		X	+	0	+	+	+
	Thermosonic		X		−	0	+	+	0
	Laser Ultrasonic		X		−	0	+	+	−
	High temperature transducers			X	+	0	+	+	−
	EMAT		X		−	0	+	+	0
Temperature measurements	Pyrometry	−		X	−	+	+	+	−
	Infrared thermography	Creep LCF Corrosion Erosion Oxidation	X	X	−	+	+	+	−
	Phosphor thermometry	−		X	+	−	0	0	−
	Induction thermography	Crack	X		−	+	+	+	−
	AE	Creep Crack		X	−	0	0	-	+
	Mm-waves	Crack Wear	X	X	−	0	+	+	−
	Pressure measurements	Crack Creep Rubbing		X	+	+	+	+	+
	DWTS	Crack		X	+	+	0	+	−
	UCTS	−		X	+	+	0	+	−
	Performance monitoring	Cracks Wear		X	+	0	−	−	+

“+” means positive and “−“ means negative in this context. “0” means neutral.
